# Biogeochemical Processes and Microbial Dynamics Governing Phosphorus Retention and Release in Sediments: A Case Study in Lower Great Lakes Headwaters

**DOI:** 10.1007/s00267-023-01859-0

**Published:** 2023-07-28

**Authors:** Nicholas Falk, Ian G. Droppo, Ken G. Drouillard, Christopher G. Weisener

**Affiliations:** 1 Flinders Accelerator for Microbiome Research, College of Science and Engineering, Flinders University, Sturt Rd, Adelaide, SA 5042 Australia; 2 Great Lakes Institute for Environmental Research, University of Windsor, 401 Sunset Avenue, Windsor, ON N9B 3P4 Canada; 3 Canada Centre for Inland Waters, Environment and Climate Change Canada, 867 Lakeshore Rd, Burlington, ON L7R 4A6 Canada

**Keywords:** Phosphorus, Nitrogen, Sediment, Headwaters, Microbial Community, Redox

## Abstract

The ability of headwater bed and suspended sediments to mitigate non-point agricultural phosphorus (P) loads to the lower Great Lakes is recognized, but the specific biogeochemical processes promoting sediment P retention or internal P release remain poorly understood. To elucidate these mechanisms, three headwater segments located within priority watersheds of Southern Ontario, Canada, were sampled through the growing season of 2018–2020. The study employed equilibrium P assays along with novel assessments of legacy watershed nutrients, nitrogen (N) concentrations, sediment redox, and microbial community composition. 20-year data revealed elevated total P (TP) and total Nitrogen (TN) at an inorganic fertilizer and manure fertilizer-impacted site, respectively. Overall, sampled sites acted as P sinks; however, agricultural sediments exhibited significantly lower buffering capacity compared to a reference forested watershed. Collection of fine suspended sediment (<63 µm) through time-integrated sampling showed the suspended load at the inorganic-fertilized site was saturated with P, indicating a greater potential for P release into surface waters compared to bed sediments. Through vertical microsensor profiling and DNA sequencing of the sediment microbial community, site-specific factors associated with a distinct P-source event were identified. These included rapid depletion of dissolved oxygen (DO) across the sediment water interface (SWI), as well as the presence of nitrate-reducing bacterial and ammonia-oxidizing archaeal (AOA) genera. This research provides valuable insights into the dynamics of P in headwaters, shedding light on P retention and release. Understanding these processes is crucial for effective management strategies aimed at mitigating P pollution to the lower Great Lakes.

## Introduction

Freshwater environments worldwide are impacted by non-point pollution of agricultural phosphorus (P) (Withers et al. 2014), with loads from croplands estimated to range from 10.4–26.4 Tg P yr^−1^ (Liu et al. 2008; Quinton et al. 2010; Yuan et al. 2018). Watersheds of the American/Canadian lower Great Lakes’ Huron-Erie Corridor (HEC) are no exception, with nutrient loadings often correlating with the number and extent of cyanobacterial blooms and water column anoxia in both Lake Erie and Lake St. Clair (Michalak et al. 2013; Kane et al. 2014; Winter et al. 2015). The St. Clair, Thames, and Detroit River have been identified as the primary water courses that channel P through the HEC (Bocaniov et al. 2019), however, the nature of the upland sources of P to these rivers is not well documented. Models have shown that a large proportion of applied terrestrial fertilizer P is retained in Canadian-side HEC watersheds as a legacy reservoir (Van Staden et al. 2021), of which an unidentified amount resides within headwater stream sediments. Addressing the stability and cycling of in-stream sediment P can help inform management practices and aid in predicting and abating nutrient loading to downstream water bodies (Zhang et al. 2019).

The potential for stream sediments to retain or release P at a given time and place (i.e., it’s P buffering capacity) can be assessed by isotherm methods which measure soluble reactive P sorption/desorption to/from the sediment matrix (Lucci et al. 2010). Various factors have been shown to influence the effectiveness of sediment P buffering. Long-term nutrient loading can saturate bed sediment with P, diminishing its retention ability over time, and change its status from a P sink to source (Chen et al. 2018). Non-point N in headwaters can also affect the fate of P since both are primary macro nutrients for aquatic biota and have a linked stoichiometry (Dodds and Smith 2016). In addition, microbes play a crucial role in N and P biogeochemical cycling in lotic systems by influencing sediment redox, which can alter nutrient speciation and stability (Mooshammer et al. 2017; Wang et al. 2022). Despite knowledge of these individual processes influencing headwater sediment P buffering, their interactions are still unspecified. Site-specific case studies that gauge sediment P buffering as a factor of nutrient legacies, N cycling, and sediment microbial metabolism/redox can supplement models that seek to classify headwaters as semi-permanent sinks or vulnerable sources of P to the lower Laurentian Great Lakes. Furthermore, the P-buffering potential of suspended sediments is often uncharacterized, despite its high affinity for dissolved P (Sandström et al. 2020). Collecting and reporting the P buffering capacity of suspended sediments in addition to benthic sediments can provide a more comprehensive estimate of in-stream P cycling.

This study assessed the bed and suspended sediment P buffering capacity of three HEC headwater segments through the growing seasons of 2018–2020. This assessment was compared with investigations of past nutrient loads using historical water quality data, as well as current measurements of sediment redox, N speciation, water quality parameters, and sediment microbial community composition. The study aimed to classify the headwater sediments as potential sinks or sources of P over space and time, identify variables that correlate with the release of sediment P, and investigate links between sediment P release and novel measurements of in-situ sediment redox profiling and microbial community turnover. By assessing a multitude of variables over time in differently impacted headwater segments, the study sought a better understanding of sediment P-buffering and the biogeochemical factors that can contribute to P storage/release in lower Great Lakes headwaters.

## Methods

### Site Selection and Watershed Characteristics

Three watercourses that drain into the HEC in southern Ontario were sampled in 2018–2020; Big Creek (BC) in Essex County, Nissouri Creek (NC) in Oxford County (both tributaries of the Thames River), and the Saugeen River (SR) in Grey County (outlets to Lake Huron). The BC watershed is flat (average slope 0.4 m km^−1^) characterized by clay loam soils (Brookston Clay) and is dominated by crops of soybean, wheat, and corn, and inorganic/chemical fertilizer amendment. The NC watershed has higher slopes (0.9 m km^−1^) characterized by loam/silt loam soils (Guelph Loam) and is dominated by pasture, corn, soybean, wheat, and hay, with a higher density of beef, dairy, and swine livestock operations, and higher production and use of manure fertilizer. Both BC and NC have been incorporated into past and ongoing non-point source nutrient monitoring initiatives and are considered representative of prevailing sub-watershed agricultural practices (Falk et al. 2021; Nelligan et al. 2021; Gospodyn et al. 2023). SR represents a more natural baseline watercourse devoid of direct agricultural influences, with higher average slopes in the drainage basin (7 m km^−1^), characterized by sandy, well-drained loam soils (Sargent Loam, Harriston Loam, Pike Lake Loam) with a higher percentage of watershed forested land (35%) than BC and NC (<10%). The Saugeen River thus represents a regional reference headwater system to compare against the inorganic/chemical and manure fertilizer-impacted sites. General climate and drainage basin data, and additional watercourse characteristics can be found in Supplementary Table S1.

### Past TN and TP Site Trends

Past Total Phosphorus (TP) and Total Nitrogen (TN) concentrations were evaluated through historical data from the Ontario Provincial (Stream) Water Quality Monitoring Network (PWQMN). Data from 2000–2020 was obtained from the publicly accessible open data catalog provided by the Ministry of Environment, Conservation and Parks (MECP) (https://data.ontario.ca/dataset/provincial-stream-water-quality-monitoring-network). Station IDs 04001303302 and 08012305702 were used for Big Creek and the Saugeen River, respectively. For the Nissouri Creek watershed, station 04001304102 (Middle Thames River) was used as a substitute, as data for Nissouri Creek was not available. The Middle Thames station is situated 14 km downstream from the Nissouri Creek sampling site within the same sub-watershed.

### Point Sampling of Bed Sediment and Surface Water

Point sampling was done at BC only in 2018, and for all three sites in 2019 and 2020. Samples were collected in the growing season, and timepoints were classified as early spring (ESP), mid spring (MSP), early summer (ES), mid summer (MS), late summer (LS), or fall (F). Only MS and F were sampled in 2020. Timepoints were selected when preceding weekly precipitation was low to capture conditions of baseflow. In this way, the effects of external nutrient loading from the surrounding catchment on bed sediments could be minimized and observations could be based on in-stream processes. However, local precipitation data was recorded for post-hoc analysis from the nearest weather station (Supplementary Fig. S2) using the Government of Canada Historical Data Archive (Government of Canada 2022). At each point sampling, the same access point was visited, with sites varying in depth from 0.5 to 1 m. Surface water was collected from the middle of the watercourse roughly 20% below the surface in high-density polyethylene (HDPE) bottles in triplicate, with bottles being rinsed thoroughly in the same waterbody beforehand and with no head space to limit oxygen diffusion. Water temperature, dissolved oxygen (DO), and oxidation-reduction potential (ORP)) were measured using a YSI EXO2 Multiparameter Water Quality Sonde. Bed sediment samples were collected in triplicate from the upper 2 cm of the sediment-water interface using shallow cores and stored in 200 mL polyethylene containers. Both water and bed sediment samples were stored on ice until the end of the sampling day, after which they were stored at 4 °C if analysis was done within 24 h, or at –20 °C if analyses were postponed for >24 h.

### Suspended Sediment Collection

To collect suspended sediment, sediment traps were placed at sites in 2019 and 2020. Phillips Tube (PT) samplers were used, which consist of hollow poly-vinyl chloride (PVC) tubes approx. 1 m in length and 10 cm in diameter (volume = 7.54 L) sealed with a threaded conical head and flat end plug with 4 mm diameter openings for fluid flow-through. The design allows for collection of the fine suspended sediment phase (<63 µm particle size) from the water column in larger quantities than possible from point grab samples (Phillips et al. 2000). For 2019, Big Creek and Nissouri Creek PT samplers were first deployed in mid spring and collected at each subsequent point sampling, thus representative suspended sediment was collected during the intervals mid spring-early summer (MSP-ES), early summer-mid summer (ES-MS), mid summer-late summer (MS-LS), and mid summer-fall (MS-F). For the Saugeen River, the mid spring (MSP) sampling timepoint was not conducted in 2019. For 2020, PT samplers were deployed over the single interval between mid summer to fall (MS-F). PT samplers were secured above the watercourse bed with the accumulated water-suspended sediment slurry collected at each point sampling in 20-gallon buckets, and the samplers cleaned and returned to their position in the watercourse after collection (Falk et al. 2022). PT sampler sediment was stored in the same way as water and bed sediment before analysis.

### Physico-Chemical Analyses of Water and Sediment

#### Select Water Chemistry

Surface water and PT sampler suspended sediment slurries were measured for total suspended solids (TSS) and for bioavailable P as soluble reactive phosphorus (SRP). TSS (mg L^−1^) was calculated by filtration of a recorded volume of water through pre-weighed 0.45 µm cellulose acetate filters and massing of filters after drying for 24 h at 100 °C. SRP was measured, after filtration in the field (0.22 µm cellulose acetate filters), on a SmartChem 170 Direct Read Discrete Analyzer via the phosphomolybdenum blue method, using ammonium molybdate, potassium antimony titrate as a catalyst, and ascorbic acid as the reducing agent. TP and TN were measured by colorimetry after digestion in sulfuric acid and potassium persulphate (Method ref. no. E3116), with analyses done by the Ontario Ministry of the Environment, Conservation and Parks (OMECP) Laboratory Services Branch. Additional analytical methods for ammonia/ammonium-N (NH_3_ + NH_4_^+^), nitrate-N (NO_3_^−^), and nitrite-N (NO_2_^−^) (method code E3364A) can be found in (Abbey et al. 2009).

#### Sediment Phosphorus Equilibrium

Estimates of bed sediment and suspended sediment P buffering capacity across timepoints were done by the Zero Equilibrium Phosphorus Concentration (EPC_0_) assay following the sorption isotherm method (Falk et al. 2021). In brief, individual 5 gram sub-samples of bed sediment were spiked with increasing initial concentrations of SRP (SRP_ini_) (4, 15, 30, 70, 150, 500, 2000 µg L^−1^) with a 0.0005 M CaCl_2_ background solution (total volume of 40 mL) and placed on an orbital shaker for 24 h. Treatments were then centrifuged (2500 × *g*, 15 min) and the supernatant removed, filtered (0.22 µm cellulose acetate), and measured for the final SRP concentration (SRP_fin_). The amount of P-sorbed per gram of sediment for each spike was calculated as:

1 $${{{\mathrm{P}}}}_{{{{\mathrm{sorb}}}}}({{{\mathrm{\mu g}}}}\,{{{\mathrm{g}}}}^{ - 1}) = \frac{{\left( {{{{\mathrm{SRP}}}}_{{{{\mathrm{ini}}}}} - {{{\mathrm{SRP}}}}_{{{{\mathrm{fin}}}}}} \right) \times {{{\mathrm{V}}}}\left( {{{\mathrm{L}}}} \right)}}{{{{{\mathrm{mass}}}}\left( {{{\mathrm{g}}}} \right)}}$$

To calculate EPC_0_ values (µg L^−1^), SRP_fin_ values were regressed against P_sorb_ values on an x–y coordinate system using a linear or Freundlich model depending on the higher r^2^. SRP_fin_ at P_sorb_ = 0 (x-intercept) of the resulting regression was calculated as the EPC_0_ value, i.e., the concentration of SRP where zero net sorption to/from the sediment occurs after the 24 h incubation, or where SRP_fin_ = SRP_ini_. For PT sampler suspended sediment, the same methodology was used except that 35 mL of the suspended sediment-water slurry was spiked rather than the 5 g of sediment. This was due to the difficulty in removing the fine sediment load from suspension. Thus, SRP_ini_ was calculated as the sum of the spiked concentration and the initial SRP concentration of the sediment-water slurry, and the slurry TSS concentration (measured identical to water sample TSS) was used to calculate the suspended sediment mass (normalized for volume of the sample). The background CaCl_2_ solution was used to make up the final 40 mL volume, with all dilution effects accounted for in calculations of EPC_0_ values. Assays were done in duplicate. Sediments are considered to be in equilibrium with respect to surface water dissolved P if the difference between measured surface water SRP and EPC_0_ does not exceed 20% (Jarvie et al. 2005; Weigelhofer et al. 2018). This criterion is used here to define site bed and suspended sediments as sinks (SRP > EPC_0_ by 20%) or sources (SRP < EPC_0_ by 20%) of SRP, or in equilibrium (SRP within 20% of EPC_0_).

#### In-situ Sediment Redox Characteristics

To assess in-situ bed sediment dissolved oxygen (DO) and oxidation-reduction potential (ORP) at sites, Unisense microsensors/microelectrodes were used with a motorized vertical field MicroProfiling system and Field Microsensor Multimeter (FMM) (https://unisense.com/). Sensors were glass-type with 100 µm tips (OX-100 and RD-100 for DO and ORP, respectively) and were calibrated in the field before profiling. For field measurements, the MicroProfiler was adhered to the linear stage and hand pressed firmly into an undisturbed section of sediment no farther than 2 meters from where bed sediment was collected. The sensors were mounted to the motor stage at an initial position approximately 1 cm above the sediment water interface (SWI). Duplicate vertical profile sequences were 2–3 cm in depth with measurements recorded every 0.3 mm. Sensors were programmed with a measure and stabilization period of 3 s, with all measurements logged on the FMM. Rates of DO and ORP change over the SWI were calculated by dividing the variable difference (µmol for DO and mV and for ORP) by the depth interval (mm). Profiles were conducted in 2019 and 2020 at the timepoints outlined in Supplementary Table S2.

### Bed Sediment Microbial Community Analysis

Bed sediments collected across the three sites in 2019 were extracted for total DNA using the QIAGEN DNeasy PowerLyzer PowerSoil Kit following the manufacturers instructions. Forward V5F (5–ATTAGATACCCNGGTAG-3) and reverse V6R (5–CGACAGAGCCATGCANCACCT-3) primers were used for amplification of a roughly 300 base pair (bp) segment of the hypervariable 16S bacterial rRNA gene for taxonomic identification using the SILVA ribosomal RNA database (Silva release 132_99_16S) (Quast et al. 2013). Sequencing was completed on an Ion Torrent Personal Genome Machine (Life Technologies) and resulting reads were processed using the Quantitative Insights into Microbial Ecology (QIIME) pipeline, QIIME2 ver. 2019.1 with details of filtering, alignment, and phylogenetic tree construction outlined previously (Falk et al. 2022). Principal coordinate analysis (PCoA) was used to visualize Bray-Curtis distances between 2019 samples with beta diversity dissimilarity tested via PERMANOVA with 999 permutations. Linear discriminant analysis Effect Size (LEfSe), a method designed for metagenomic data, was used to further identify significant biomarker taxa across sample groups of interest (Segata et al. 2011).

## Results

### Past PWQMN TN and TP Concentrations at Sampling Locations

**Fig. 1:**
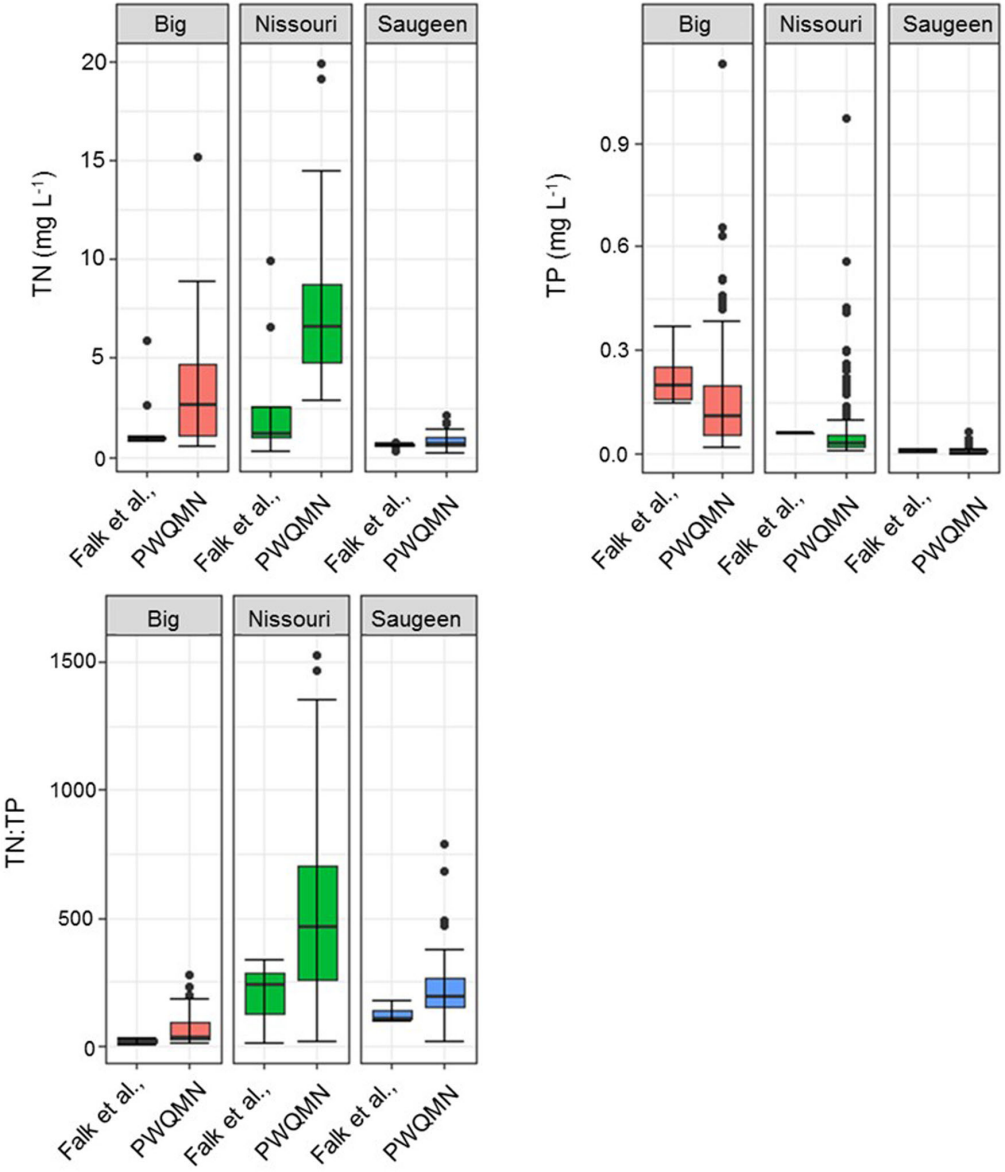
Total Nitrogen (TN), Total Phosphorus (TP), and TN:TP for Big Creek, Nissouri Creek, and the Saugeen River. Falk et al., data represents the range of values collected as part of this study, with Provincial Water Quality Monitoring Network (PWQMN) data representing values obtained for 2000–2020 from the open data catalog from the Ministry of Environment, Conservation and Parks (MECP)

Surface water TN, TP, and TN:TP ratios were analyzed for point sampling and from PWQMN historical data available from 2000–2020. Trends are displayed by variable, broken down by site in Fig. 1.

### Bed and Suspended Sediment Buffering Capacity by EPC_0_

**Fig. 2:**
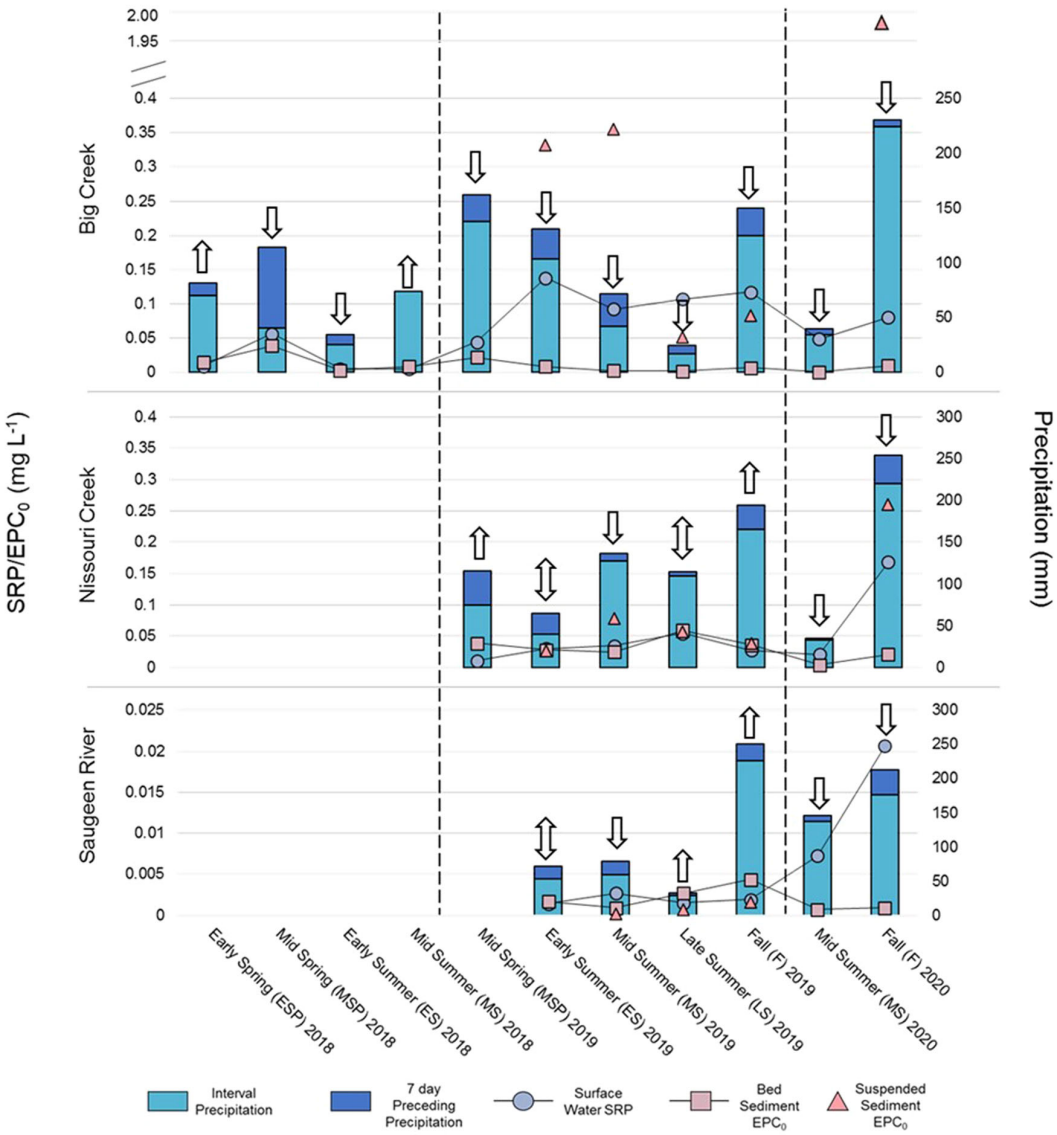
Seasonal point sampling characteristics for Big Creek, Nissouri Creek, and the Saugeen River for 2018 through 2020. Stacked bars represent the total interval precipitation (mm) with top sections specifying the seven-day precipitation amount preceding sampling. Dashed lines separate sampling years: Nissouri Creek and the Saugeen River were not sampled in 2018. Upward arrows indicate bed sediment P-sources, downward arrows indicate P-sinks, and double-sided arrows represent P-equilibrium

Point sampling values of SRP, bed and suspended sediment EPC_0_, and preceding sampling precipitation are shown in Fig. 2, with annual averages for SRP and EPC_0_ per site outlined in Supplementary Table S3.

Bed sediments at Big Creek in 2018 were defined as P-sources in Early Spring and Mid Summer, and P-sinks in Mid Spring and Early Summer. In 2019 and 2020, all SRP concentrations exceeded bed sediment EPC_0_ values by >20% at Big Creek and were defined as P-sinks. Nissouri Creek bed sediments were defined as P-sources in Mid Spring and Fall 2019, P-sinks in Mid Summer 2019 and all of 2020, and in equilibrium in Early Spring and Late Summer 2019. Mid Spring 2019 was a notable high P-source designation with EPC_0_ exceeding SRP by 246% (0.039 and 0.011 mg L^−1^ for EPC_0_ and SRP, respectively). Saugeen River surface water SRP and bed EPC_0_ concentrations were lower than both BC and NC and defined as P-sources in Late Summer and Fall 2019, P-sinks in Mid Summer 2019 and all 2020, and in equilibrium in Early Summer 2019.

Suspended sediment EPC_0_ values at Big Creek were significantly higher than bed sediments, with values of 0.33, 0.35, 0.05, and 0.08, and mg L^−1^ for Mid Spring-Early Summer (P-source), Early Summer-Mid Summer (P-source), Mid-Summer-Late (P-Sink) Summer, and Late Summer-Fall (P-sink) intervals, and 1.9 mg L^−1^ for the Mid Summer-Fall interval of 2020 (P-source). For Nissouri Creek, suspended sediment EPC_0_ values were lower and closer to bed sediment equilibrium concentrations, measuring 0.03, 0.08. 0.06, 0.04 mg L^−1^ for the 2019 intervals (in chronological order) and 0.26 mg L^−1^ for the 2020 interval. Nissouri Creek suspended sediment was deemed a P-source for the Early Summer-Mid Summer 2019 interval and Mid Summer-Fall 2020 interval. Saugeen River exhibited lower EPC_0_ values for suspended sediment than bed sediment for all intervals, measuring 1.55 × 10^−4^, 6.96 × 10^−4^, and 0.0016 mg L^−1^ for Early Summer-Mid Summer, Mid Summer-Late Summer, and Late Summer-Fall 2019. All intervals for SR suspended sediment were defined as P-sinks. The Saugeen River PT sampler was dislodged during deployment in 2020, thus no suspended sediment data was retrieved. Additional PT sampler deployment information can be found in Supplementary Table S10.

### Vertical Microsensor Redox and DO Profiling

**Fig. 3:**
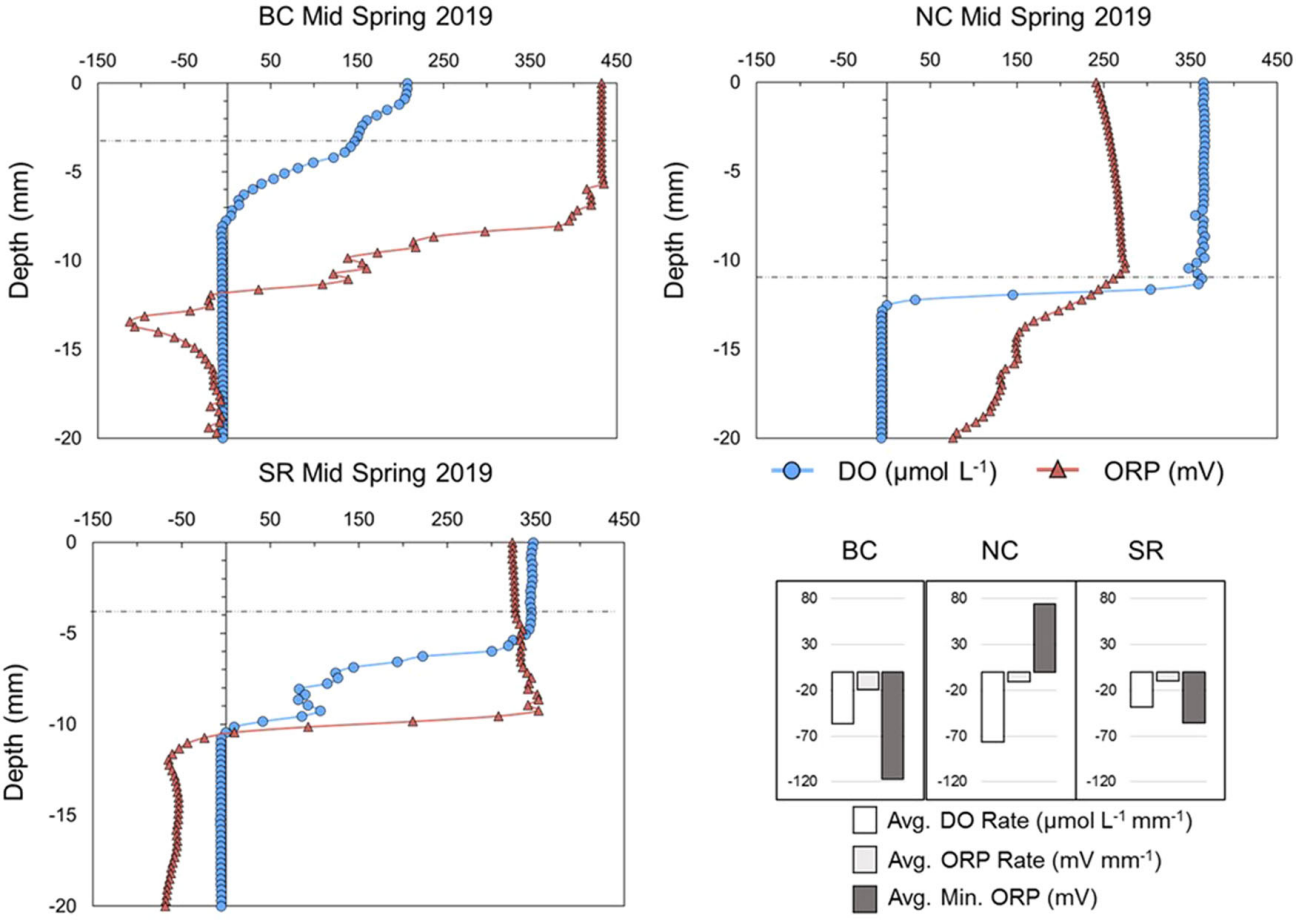
Mid Spring 2019 vertical microsensor profiles for dissolved oxygen (DO) and oxidation-reduction potential (ORP). Horizontal dashed lines represent the location of the sediment water interface (SWI). Inset bar graph shows average DO and ORP depletion rates with depth, and average minimum (Min) recorded ORP across all measurements for Big Creek (BC), Nissouri Creek (NC), and the Saugeen River (SR)

Vertical microsensor profile data is summarized in the inset bar chart in Fig. 3, with average values provided for DO rate, ORP rate, and Min ORP calculated for each site from all measurements over 2019–2020. Average DO rates were −56.4, −76.2, and −38.3 µmol L^−1^ mm^−1^ over the SWI for Big Creek, Nissouri Creek, and the Saugeen River, respectively, with ORP rates of −19.1, −10.3, and −9.9 mV mm^−1^. Average Min ORP values were −116.9, 75.6, and −55.6 mV for Big Creek, Nissouri Creek, and Saugeen River bed sediments. Sample vertical profiles from Mid Spring 2019 for each location are shown in Fig. 3 which were characteristic of site trends observed through the study.

### Temporal and Spatial Bed Sediment Microbial Community Analysis

**Fig. 4:**
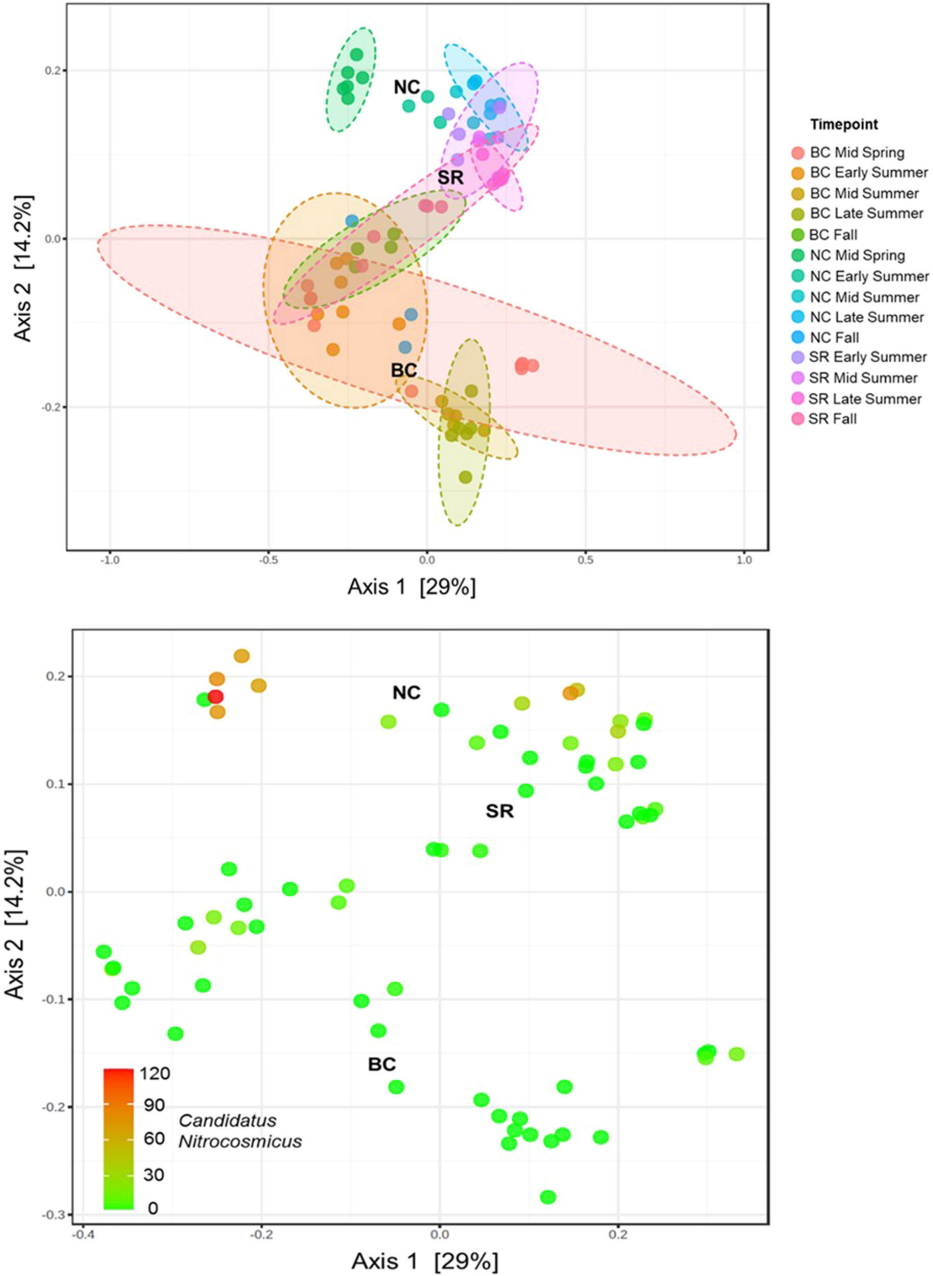
PCoA with Bray–Curtis distances of genus-level microbial community composition across samples. **Top panel:** Samples labeled by site and timepoint with 95% confidence ellipses. BC Big Creek, NC Nissouri Creek, SR Saugeen River. **Bottom panel:** PCoA overlain with a heatmap of the taxon abundance of the ammonia oxidizing archaea (AOA) *Candidatus Nitrosocosmicus*

**Fig. 5:**
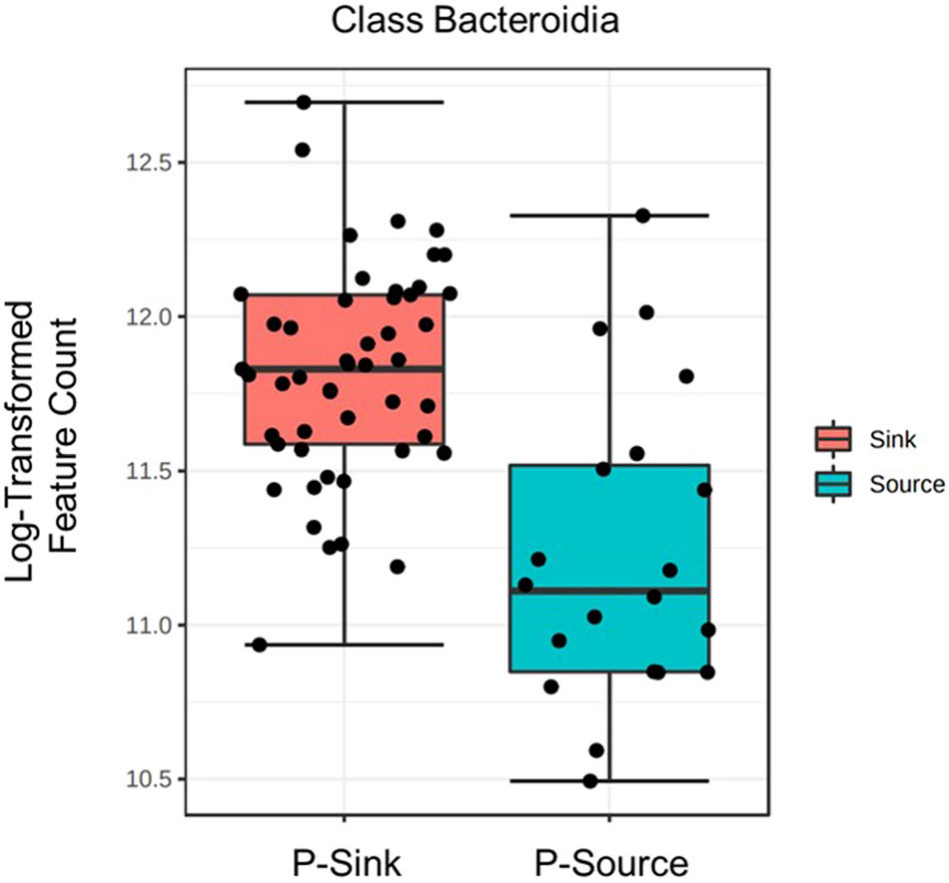
Log-transformed count comparison of Bacteroidia in phosphorus sink (P-sink) and phosphorus source (P-source) sediment across all study samples. Linear discriminant analysis Effect Size (LEfSe) False detection rate (FDR) adjusted *p*-value = 0.0010953

Microbial community DNA sequencing yielded 69 samples for 2019 bed sediments, with an average count of 37,676 sequence per sample, resulting in identification of 454 microbial features after filtering. Ordination of samples by PCoA using Bray–Curtis distance is displayed in Fig. 4A, with the first 2 axes explaining 29% and 14.2% of the variation in the dataset. Nissouri Creek and Saugeen River communities showed greater similarity to each other than to Big Creek communities in the resulting ordination, with Big Creek samples showing higher inter-sample variation. The Mid Spring timepoint for Nissouri Creek showed a radiation towards the top left quadrant of the ordination. Biomarker analysis by LEfSe was applied to this timepoint and was significantly abundant in the phyla Pseudomonadota (formerly Proteobacteria), Actinobacteria, Firmicutes, and Thaumarchaeota. Biomarker genera from this timepoint are listed in Supplementary Table S4 and included the Ammonia Oxidizing Archaea (AOA) *Candidatus Nitrosocosmicus*, and the denitrifiers *Dechloromonas*, *Rhodoferax*, and *Thauera*. Figure 4B displays the abundance of *C. Nitrosocosmicus* across samples, highlighting its strong association with Nissouri Creek Mid Spring samples. The class Bacteroidia was the only taxonomic feature found to be a significant bioindicator of P-sink sediments across all samples (Fig. 5), with no significant bioindicators of P-source events observed.

### Site Physico-chemical Water Quality Variable Correlations

We tested water quality and sediment redox variables for pair-wise correlation analysis via Spearman’s Rank test. Significant correlations are displayed in Table 1 and all results in Supplementary Table S5. Surface water SRP was positively correlated with surface water TP (rho 0.94, *p* < 0.05) with no correlations to sediment redox parameters. Surface water TP was also correlated with surface water DO (rho −0.97, *p* < 0.05), surface water TSS (rho 0.83, *p* < 0.05), and minimum (Min) sediment ORP (rho −0.76, *p* < 0.05). Bed sediment EPC_0_ was slightly but significantly correlated to surface water NO_3_ and bed sediment DO depletion rate. Study-wide sediment and water quality data are reported in Supplementary Table S6.

**Table 1:** Significant sediment and water quality variable correlations

Variables		*P*-value	Statistic ρ (rho)
TP	SRP	5.48E-05	0.93939
TP	DO	0.000165	−0.96667
TSS	DO	0.000795	−0.78901
Min ORP	ORP Rate	0.001637	0.85455
TSS	TP	0.00294	0.8303
NO_3_	EPC_0_	0.005801	0.65588
Precip Pre	ORP	0.007559	0.62293
NO_3_	DO	0.012593	−0.69231
Min ORP	DO	0.021776	0.80952
Temp	ORP	0.024715	−0.54167
Min ORP	TP	0.036756	−0.7619
DO rate	EPC_0_	0.04254	−0.64848

*TP* surface water total phosphorus, *SRP* surface water soluble reactive phosphorus, *DO* surface water dissolved oxygen, *Min ORP* bed sediment minimum oxidation-reduction potential, *OPR rate* bed sediment oxidation-reduction potential rate, *Precip Pre* 7-day precipitation total preceding sampling, *TSS* surface water total suspended solids, *NO*_*3*_ surface water nitrate, *EPC*_*0*_ bed sediment zero equilibrium phosphorus concentration,*Temp* surface water temperature

## Discussion

### Headwater Bed Sediment P Buffering

Big Creek and Nissouri Creek sediments had higher values of SRP and EPC_0_ than the less agriculturally-impacted reference Saugeen River. Waterbody sediments affected by non-point P sources commonly accumulate legacy P (Palmer-Felgate et al. 2009), and it’s confirmed here that the sites with higher present and historical levels of surface water TN and TP also exhibited reduced sediment P buffering potential as measured by zero phosphorus equilibrium tests. However, the range of EPC_0_ values observed across the three years of data collection (0.7–60 µg/L) were in the lower end of other studies of similar non-point P lotic systems (4–183 µg/L) (Falk et al. 2021). This suggests that although 25% of bed sediments sampled were defined as P-sources (i.e., EPC_0_ > SRP by 20%) the magnitude of these internal release events is relatively moderate. Temperature and precipitation did not show any significant correlations with bed sediment EPC_0_ or surface water SRP, and P-source designations occurred more during spring and fall timepoints than during the summer. Thus, there was no evidence for higher EPC_0_ values or sediment P source events occurring because of dryer, warmer conditions, as has been suggested in other studies (Zhang et al. 2015; Stutter et al. 2021). In addition, the study design called for sampling when the preceding weekly precipitation was lower than seasonal averages to capture baseflow periods that often exhibit enhanced sediment P-desorption (Simpson et al. 2021), yet still only several P-source events were recorded. Thus, it was confirmed that headwater bed sediments studied here behaved primarily as P-sinks (75% of observations), and that the Saugeen River represents an appropriate reference baseline compared to agriculturally influenced systems. However, observations at Big Creek and Nissouri Creek revealed novel site-specific factors that may contribute to sediment P desorption and potential loading downstream, which are discussed below.

#### Non-point P from Suspended Sediment at Big Creek

Despite bed sediments at Big Creek showing effective capacity to buffer in-stream P concentrations, water column SRP and TP concentrations were higher on average than Nissouri Creek. This suggests that P at Big Creek originates externally. External non-point P at Big Creek could be from a combination of sources. Primarily, there is a large extent of tile-drainage in the BC watershed due to high clay soils, and tile drains have been shown to act as conduits of soil P to waterbodies (King et al. 2015). Tile drain effluent was collected in Mid Spring 2019 at BC when tiles were draining into the watercourse, with measured SRP concentrations of 0.07 mg L^−1^ (Supplementary Table S6), which was greater than the in-stream concentration of 0.04 mg L^−1^ at the time, offering evidence that tiles contribute to high SRP at Big Creek. However, more observations from tile drains across southern Ontario watershed are necessary to make this connection.

Another probable source of P at Big Creek is from suspended sediment. TSS concentrations were high at BC, likely due to the high clay content of the surrounding soils, with fine-grained sediment less likely to deposit in lotic systems and remain in suspension. Correlations across sites showed that surface water TSS and TP were significantly correlated (rho 0.83, *p* < 0.05), as were SRP and TP (rho 0.94, *p* < 0.05). In addition, determination of suspended sediment EPC_0_ values showed that the fine particulate phase could be a much greater accumulator and source of SRP than bed sediments, and that in many cases, suspended sediment at Big Creek was at or near saturation with respect to dissolved P. For example, suspended sediment EPC_0_ values at Big Creek were found to be as high as 200 times greater that corresponding bed sediment values, and suspended sediment collected during the 2020 interval was measured to have an EPC_0_ over 2000% greater than corresponding surface water SRP, constituting a major internal P-source. It’s possible that suspended sediment is an overlooked source of internal P in suspension at Big Creek, as has been the case in other regional watersheds of the lower Great Lakes (Shinohara et al. 2018; Markovic et al. 2020).

Fine suspended sediment (<63 µm) has higher affinity for dissolved species compared to coarser benthic sediments due to the high surface area to volume ratio of particles (Wang et al. 2006; Agudelo et al. 2011; Droppo et al. 2015). However, it is also possible that the exceedingly high suspended sediment EPC_0_ values observed originate from the methodology (i.e., PT time-integrated samplers). Specifically, suspended sediment EPC_0_ values appeared to increase with longer deployment and with higher TSS accumulations, even after mass-normalization corrections. Therefore, it is possible that additional SRP is being adsorbed to sediment within the PT sampler than would occur in the natural stream setting, and the collected fine suspended sediment is behaving as an SRP filter. This could explain the SRP saturation effects and high saturation seen in other studies that use this methodology (Emelko et al. 2016). In addition to SRP filter effects, the redox state of the PT samplers can also confound measurements of redox-sensitive species (such as P adsorbed to Fe and Mn-oxy/hydroxides) (Falk et al. 2022). To better evaluate suspended phase EPC_0_ values, sampling methods that can obtain appropriate sediment masses (such as point centrifugation) can be paired with observations from time-integrated PT samplers. Despite these drawbacks, suspended sediment as a source of dissolved P should not be ignored in agricultural catchments, as the shorter collection intervals in this study still suggest very high P-desorption potentials from this phase. Estimates here show that if particulate phosphorus is transported to receiving waterbodies with lower SRP concentrations, the magnitude of diffusive release of bioavailable P from fine suspended sediment in Big Creek can be over 100 times greater than from bed sediments.

#### Non-point P at Nissouri Creek from Bed Sediments

Lower TP, TSS, and suspended sediment EPC_0_ values, but higher bed sediment EPC_0_ values at Nissouri Creek suggest that the site is influenced by internal P loading mechanisms. Analysis showed a significant correlation between bed sediment EPC_0_ and vertical sediment DO depletion rate as measured by microsensor redox profiles. Thus, the more rapidly that DO decreases through the SWI, EPC_0_ values were higher. At Nissouri Creek in Mid Spring 2019, the DO depletion rate was −158.4 µmol L^−1^ mm^−1^, the greatest rate value recorded from any site, and bed sediment was determined to be a significant source of SRP (EPC_0_ > SRP by 246%). Local sediment anoxia has been shown in field and laboratory studies to increase P desorption from the sediment matrix, as reductive dissolution of oxides and hydroxides of Fe and Mn can release immediately bioavailable P-species adsorbed to these minerals (Ding et al. 2018; Casillas-Ituarte et al. 2019). P fluxes from sediments associated with these biogeochemical redox processes have been cited over a wide ORP range, from as great as 300 mV to –200 mv (Hill and Robinson 2012; Parsons et al. 2017; Ding et al. 2018). ORP microsensor profiles showed that NC sediment reached an average minimum of 110 mV in Mid Spring 2019, indicating suboxic, reducing conditions, within previous recorded ranges for microbially-mediated Fe/Mn reduction. Despite Big Creek sediments having lower average sediment ORP, previous analysis showed that Nissouri Creek had greater concentrations of labile and reducible Fe-bound sediment P, which can be more vulnerable to redox-driven desorption. Thus, there is a plausible link between higher EPC_0_ values and sediment DO/ORP characteristics at this location.

Nissouri Creek was also characterized by high N:P ratios, which could be a symptom of watershed-wide manure-application. N and P are both primary macronutrients for stream productivity and are consumed via microbial metabolism. The abundance of N-species at Nissouri Creek (Supplementary Fig. S1) may result in local P-limitation, which can cause loosely bound sediment P to desorb to the water column/pore water in the face of changing concentration gradients (McDowell et al. 2017). Thus, N speciation should also be accounted for when determining drivers of internal P sources, as it’s impact on in-stream productivity can alter P-gradients between sediments and surface waters. Excessive nitrogen inputs can also result in shifts in the composition and abundance of microbial communities, which can be detectable and informative of bed sediment redox state, as discussed below.

### Microbial Community Effects

The microbial influences on sediment redox reactions and N and P cycling are garnering attention as primary factors in nutrient stability at the SWI. Genomic investigations can detect microbial community difference between site sediments and identify the taxa that contribute most to ecological variables.

No microbial taxa were found to be inter-site indicators of P-source events. However, the class Bacteroidia was significantly more abundant in sediments that were P-sinks. Bacteroidia are widespread in aquatic environments (Tandon et al. 2021), thus the specific mechanisms that lead to their abundance in well P-buffered sediments are difficult to surmise. However, if future studies find similar conclusions about this bacterial class, they may be incorporated into indexes of stream health as indicators of well nutrient-buffered systems.

Denitrifying (*Dechloromonas*, *Rhodoferax*, and *Thauera*) and nitrifying (*Candidatus Nitrosocosmicus*) microbial taxa were significantly abundant in Mid Spring 2019 Nissouri Creek bed sediments, and N-chemistry showed lower and higher average concentrations of water column nitrate and ammonia/ammonium, respectively, at this timepoint (Supplementary Fig. S1). This time point also had the highest EPC_0_:SRP ratio within the study and was characterized as a significant P-source event. As DO depletion rates across the SWI were also shown to be the highest at Nissouri Creek, it is proposed that rapid oxygen removal not only promotes desorption of sediment P but leaves nitrate as the next favorable terminal electron acceptor for microbial metabolism, which can then be consumed by the denitrifying taxa observed (Holmes et al. 2009; Fuchs et al. 2011; Sanford et al. 2012; Weisener et al. 2017; Palacin-Lizarbe et al. 2019; Yang et al. 2019). The presence of ammonia species, which may be a product of concomitant nitrate reduction, may also be responsible for the proliferation of the AOA *C. Nitrosocosmicus*. This demonstrates a conceptual model linking the detection of denitrifying and AOA taxa to internal P-desorption potential and corroborates with previous studies that identified similar microbes in manure-applied settings (Lopatto et al. 2019; Liao et al. 2020; Ye et al. 2021). This process may be specific to manure-afflicted watersheds, as observed here, or may be more widespread, a hypothesis that was limited here due to the small number of locations but can be tested in future studies with greater sampling scopes. However, it is a valuable case study observation that is corroborated by precision in-situ redox profiling and P equilibrium measurements.

## Conclusions

Tributary loads of P entering the lower Great Lakes are often lower than estimated based on terrestrial inputs. Thus, watercourse bed and suspended sediments must play vital roles in retaining P in upland, agriculturally intensive watersheds, but the stability of this P reservoir is unknown to management efforts. This study over three years within an inorganic/chemical fertilizer-influenced, manure fertilizer-influenced, and a reference forested watercourse revealed site-specific biogeochemical mechanisms associated with in-situ sediment phosphorous release and retention and the key variables at play.

Overall, site sediments acted as P-sinks during the growing season, with the fertilized sites exhibiting higher P-desorption potentials and historical concentrations of TN and TP. The inorganic fertilizer-influenced stream showed high TP, SRP, and TSS, and had a higher risk of internal P-release from suspended sediments than from bed sediments. High clay content in surrounding soils likely promoted P-sorption to the fine suspended sediment phase, which could be poised to diffusive desorption when transported through the river continuum. The manure-influenced site was defined by higher TN, bed sediment EPC_0_ values, and DO depletion rates over the SWI. N-driven primary production at this site by algae could serve to remove SRP from the water column and promote diffusive flux of SRP from labile and reducible sediment-P phases. In addition, denitrifying and AOA microbial taxa were shown to be significant biomarkers during this P-desorption event, and could serve as bioindicators of internal P release in future assessments. The abundance of the microbial class Bacteroidia in well P-buffered sediments is another valuable observation in the application of microbial genomic tools for nutrient management.

In summary, this study provides a case study of P-retention trends in low-order, upland watercourses in southern Ontario, Canada, and revealed site-specific vulnerabilities associated with internal release of P from bed and suspended sediments by incorporating novel microsensor DO and ORP measurements and microbial community analysis. Future studies in watersheds that contrast impacts by inorganic/chemical and manure fertilizers may use the results found here as baselines, particularly where long-term nutrient histories are unknown.

### Supplementary Information

Supplementary Figure S1

Supplementary Figure S2

Supplementary Tables revised

Supplementary Figures

## Data Availability

Additional data and material available upon request.
