# Supplementary figures and images for: Biogeochemical Processes and Microbial Dynamics Governing Phosphorus Retention and Release in Sediments: A Case Study in Lower Great Lakes Headwaters
Source: Environ Manage. 2023 Jul 28;72(5):932–44. doi: 10.1007/s00267-023-01859-0 (PMC10509119; doi:10.1007/s00267-023-01859-0)

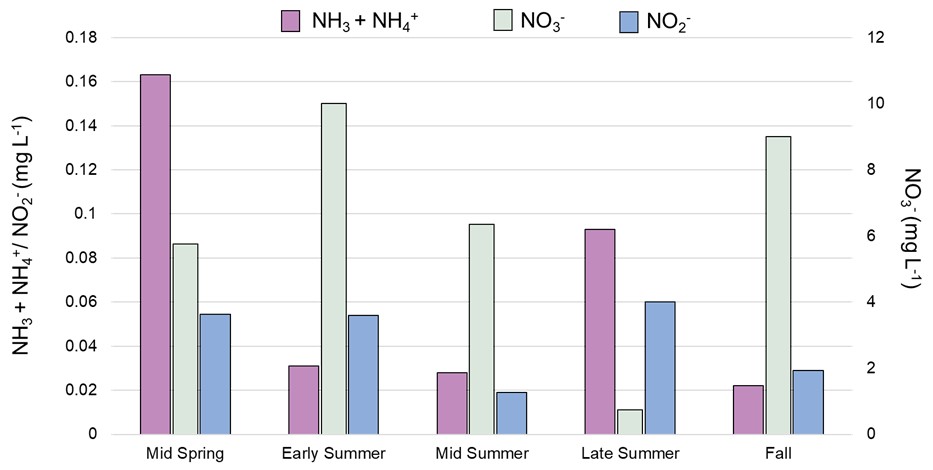

Supplement: Supplementary file 1 — Supplementary Figure S1 [file 267_2023_1859_MOESM1_ESM.jpg]

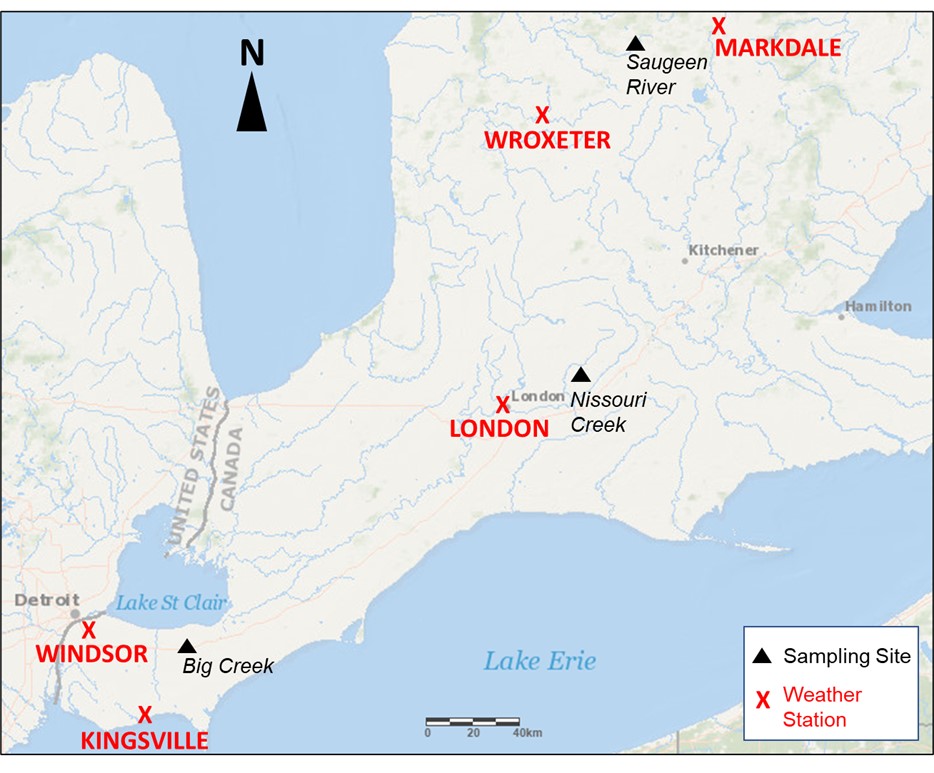

Supplement: Supplementary file 2 — Supplementary Figure S2 [file 267_2023_1859_MOESM2_ESM.jpg]
