# Supplementary material for: Biogeochemical Processes and Microbial Dynamics Governing Phosphorus Retention and Release in Sediments: A Case Study in Lower Great Lakes Headwaters
Source: Environ Manage. 2023 Jul 28;72(5):932–44. doi: 10.1007/s00267-023-01859-0 (PMC10509119; doi:10.1007/s00267-023-01859-0)
Supplement: Supplementary file 4 — Supplementary Figures [file 267_2023_1859_MOESM4_ESM.docx]

Figure S1: Nissouri Creek 2019 concentrations of ammonia-N species (NH_3_ + NH_4_^+^), nitrate-N (NO_3_^-^), and nitrite-N (NO_2_^-^).

Figure S2: Location of sampling sites and nearest weather stations where local precipitation data was found.
